# Cases Reported for the Medical Examiner

**Published:** 1862-11

**Authors:** O. B. Ormsby

**Affiliations:** Assistant-Surgeon, 18th Ill.


ARTICLE XXXVI.

### CASES REPORTED FOR THE MEDICAL EXAMINER.

By O. B. ORMSBY, Assistant-Surgeon, 18th Ill.

No. I. B., a private of the 18th Regt. Ill. Vols., aged 24 years, black hair, dark complexion, very robust, was wounded at Fort Donelson by a musket ball, which entered the anterior aspect of the left thigh, and passed directly backwards, fracturing the femur in the middle-third.

Loose fragments were removed, and cold dressings applied during three days, when he was brought in to Dover. Here, Dr. Davis, his surgeon, constructed a simple inclined plane, consisting of two straight splints of the length of the injured limb, fastened together at the foot, and having canvas tacked across the under side to act as a bed for its reception. Light bandages were thrown around the limb. Placed in this contrivance; and the patient who had been much exposed to rigorous weather, was immediately put upon generous diet, and stimulants. As there had been considerable comminution of the broken bone, immediate union was not anticipated, but the limb was maintained at rest in a horizontal position without extension, and treated by application of cold. Suppuration was established within a few days, and some pieces of bone were discharged. On the tenth day after the receipt of the injury, he was sent by steamer to St. Louis, where, under the same general plan of treatment, (that is the "let alone plan,") he continued to improve, until sufficient union had taken place to admit of his walking on crutches, when he was sent home. He is now able to walk with a cane.

No. II. W., a private of the 18th Regt. Ill. Vols. Light hair, light complexion, and blue eyes, American, aged 25 years, robust and healthy, was wounded in the battle of Fort Donelson by a minie ball, which passsed obliquely through the left thigh, breaking the bone at the junction of its upper and middle thirds. Examination revealed great comminution, and a number of fragments were removed from the wound.

Simple dressings were applied until the third day, when the limb was placed upon the straight inclined plane without extension, and dressed by the application of a light bandage around both splint and limb. The foot was raised two or three inches, so that the limb just cleared the bed, and in that position left at rest. The strength of the patient was supported, suppuration was kindly established, and on the tenth day he was transferred to St. Louis, where he remained under the same general plan of treatment, until able to travel; when he was sent home. His limb is considerably shortened, but still, more useful than any substitute with which I am acquainted

No. III. C. a private of the 18th Ill. Vols., aged 45 years, robust in appearance, but has suffered much exposure when young, was wounded at the battle of Fort Donelson by a musket ball, which passed obliquely through the left thigh, fracturing it near its middle. So great was the shock, and permanent depression following, that an operation was not deemed advisable. Stimulants, and generous diet, were freely administered; but he soon sunk into a muttering delirium: to relieve which, all efforts proved unavailing. He remained in this condition until the tenth day, when he died, no operation for his relief having been attempted. It was concluded by all who examined him, that an operation would be a gratuitous cruelty.

No. IV. G., a private of the 18th Regt. Ill. Vols., American, aged 19 years, dark complexion, medium height and size, quite active and robust, was wounded in the battle of Fort Donelson by a musket shot, which passed through the right thigh, breaking the femur at the junction of its middle and upper thirds. When brought in, he manifested considerable depression, but rallied under the use of stimulants. The bone was so badly shattered as to induce the belief that recovery without an operation could not be hoped for; and as it appeared that in consideration of his age and general good health, recovery after operation might be at least possible; amputation was decided upon. The operation, (circular), was quickly and neatly performed by his surgeon, and for a time he seemed to bid fair for recovery. Upon the second day, however, the wound assumed a lived hue, the tongue became dry, the extremities cold, and the pulse rose to 220 in the minute. Brandy, carb. ammonia, and other stimulants, were freely administered, but he became delirious, and upon the fourth day died. In the case (No. III.) preceding this, an operation would in all probability have been followed by a very similar result.

Reflecting on the cases above narrated, and others of the same character, which have come within the scope of my observation, I am led to conclude that many limbs are amputated, which might with as little, and, perhaps, even less risk to the patient's life, be saved. The difference in gravity between an injury, such as is commonly produced by the passage of a ball through the shaft of the femur, and that which is produced by the knife of the surgeon in amputation of the thigh, appears to me to be less considerable than many believe.

In either case, the wound may be considered, and should be treated as a very dangerous one. In the treatment of gunshot injuries of the thigh upon the conservative principle, I apprehend that in the first place care should be exercised to have the external wound sufficiently large for the free escape of pus, and foreign bodies, which may be lodged there. All foreign substances should of course be removed, but, upon this point military surgeons caution us (and I think very justly to), against the forcible abduction of fragments, which, although they may be entirely separated from the bone, are still adherent to the periostium, as laceration is liable to ensue to the extent of serious interference with the reproduction of bone. No doubt exists in my mind that many of these splinters (particularly in cases which result favorably), become reunited firmly again to the bone. In my opinion, the application of extension to these cases is bad practice. The wound is of so grave a character, that, under the most favorable circumstances, if the patient recover he will do well, and every source of irritation, however slight, is so much in the scale against him. In these injuries also, very frequently a considerable portion of the whole circumference of the bone is entirely broken up and removed; thus when the limb is fully extended, leaving a vacancy of perhaps an inch in the length of the bone quite vacant. If the extension is kept up, this entire space must be filled with callus, beside the envelopment of the ends of the bone. The organization of callus at such a distance from the living structure is not very readily accomplished: more time being required for its completion.

It is evident, that in order to fix the bone, and render it immovable, would, under these circumstances, require a higher degree of organization; and more complete hardening of the callus than is requisite, where the fractured surfaces are in contact, thereby consuming still more time.

By the admission of air into the cavity thus resulting, an extensive suppuration may be set up in the medullary canal, when a short time will terminate the life of your patient. Whereas, had no extension been employed, the broken ends would have been approximated by the action of the muscles; the small cavities remaining, would have been filled and protected, first, by coagula, then by lymph, or other organized matter; callus would shortly have enveloped the ends of the bone, and possibly, at last, recovery might be the result. I will only remark further, that the fractures produced by ball, very generally so nearly transverse, that little or no displacement results from muscular action; and where displacement does occur from this cause, I would employ only just so much extension as would retain the bone properly in position.

For the purpose of treating fractures, where extension is not desirable, the most simple, and for the field, the best apparatus I think to be the one used by Dr. DAVIS, as described above. One of its greatest advantages is, that materials for its construction are always at hand. A couple of pieces of board, a few nails, tacks, and a strip of canvas, or piece of tent flie, is required; or failing these, the drawer leg of your patient may be cut off, a small stick or withe bended and introduced through it, with the bow at the foot: the limb raised in it, and then swung by a cord, or raised by some other means, and left at rest. I have seen a contrivance of this kind, answer an admirable purpose, and in the absence of better appliances it is certainly worthy of trial.

